# Opportunistic soaring by birds suggests new opportunities for atmospheric energy harvesting by flying robots

**DOI:** 10.1098/rsif.2022.0671

**Published:** 2022-11-23

**Authors:** A. Mohamed, G. K. Taylor, S. Watkins, S. P. Windsor

**Affiliations:** ^1^ RMIT University, Melbourne, Victoria 3000, Australia; ^2^ Department of Biology, Oxford University, Oxford OX1 3SZ, UK; ^3^ Department of Aerospace Engineering, University of Bristol, Bristol BS8 1TH, UK

**Keywords:** autonomous flight, bird flight, dynamic soaring, static soaring, slope soaring, thermal soaring

## Abstract

The use of flying robots (drones) is increasing rapidly, but their utility is limited by high power demand, low specific energy storage and poor gust tolerance. By contrast, birds demonstrate long endurance, harvesting atmospheric energy in environments ranging from cluttered cityscapes to open landscapes, coasts and oceans. Here, we identify new opportunities for flying robots, drawing upon the soaring flight of birds. We evaluate mechanical energy transfer in soaring from first principles and review soaring strategies encompassing the use of updrafts (thermal or orographic) and wind gradients (spatial or temporal). We examine the extent to which state-of-the-art flying robots currently use each strategy and identify several untapped opportunities including slope soaring over built environments, thermal soaring over oceans and opportunistic gust soaring. In principle, the energetic benefits of soaring are accessible to flying robots of all kinds, given atmospherically aware sensor systems, guidance strategies and gust tolerance. Hence, while there is clear scope for specialist robots that soar like albatrosses, or which use persistent thermals like vultures, the greatest untapped potential may lie in non-specialist vehicles that make flexible use of atmospheric energy through path planning and flight control, as demonstrated by generalist flyers such as gulls, kites and crows.

## 1. Introduction

**Figure 1.**
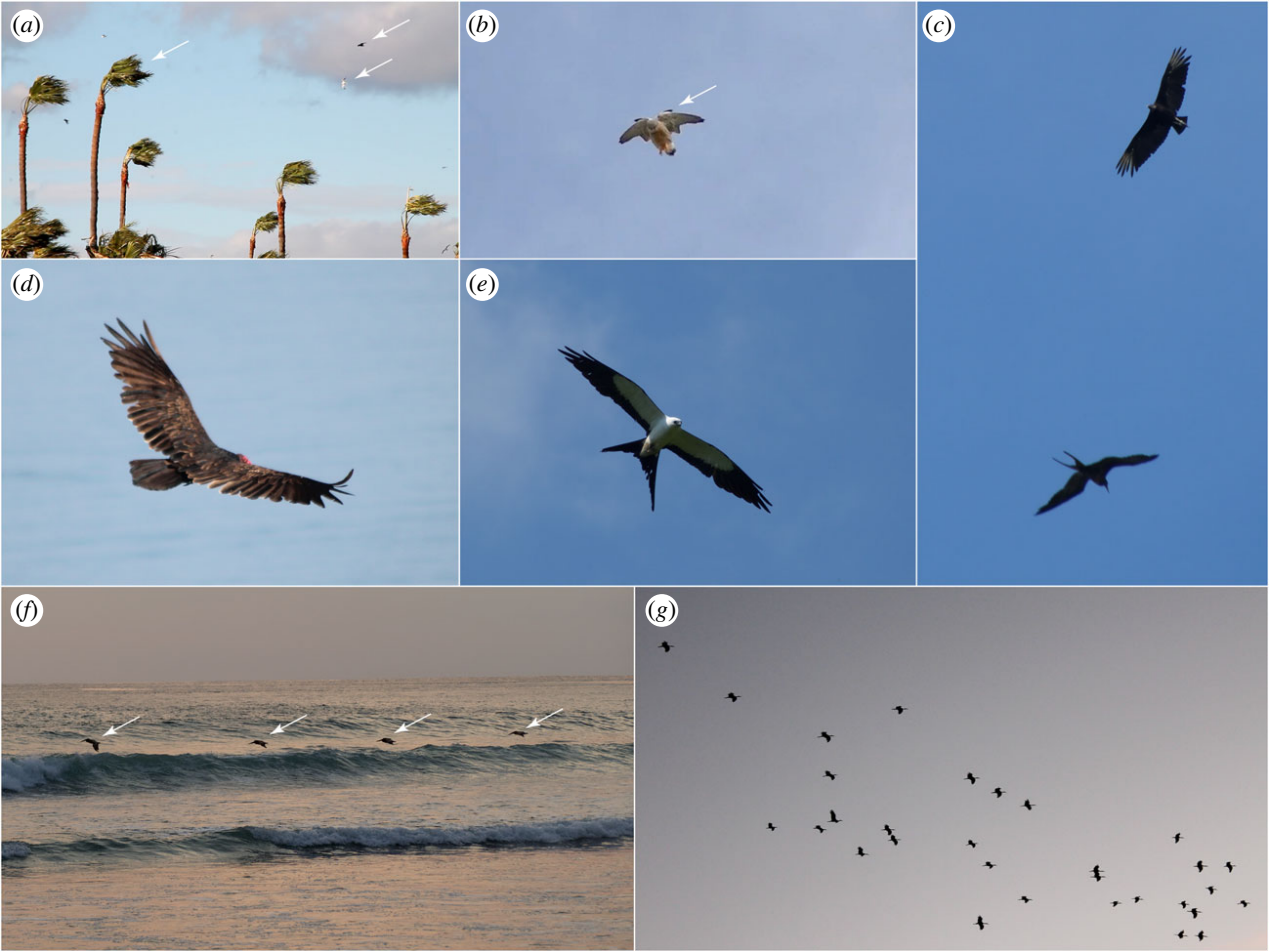
Soaring morphology and flight behaviour of birds. (*a*) Gulls *Larus* spp. and crows *Corvus* spp. soaring by combining gliding and flapping in high winds; note bending of palm trees. (*b*) Common kestrel *Falco tinnunculus* wind-hovering in strong orographic updraft; note morphing wings with alula feathers extended as a leading-edge flap; see electronic supplementary material, Movie S1. (*c*) Black vulture *Coragyps atratus* (above) and magnificent frigatebird *Fregata magnificens* (below) soaring the same thermal updraft (i.e. both birds in same photograph); note their very different flight morphologies: the frigatebird has approximately twice the aspect ratio and two-thirds the wing-loading of the vulture. (*d*) Turkey vulture *Cathartes aura* soaring an orographic updraft; note dihedral with wingtips canted upwards providing lateral stability. (*e*) Swallow-tailed kite *Elanoides forficatus* soaring low over rainforest; note forked tail used in lateral control. (*f*) Brown pelicans *Pelecanus occidentalis* performing sweeping flight along a long, steady wave in calm conditions; note the similar altitude of the four birds, sustained by the associated updraft. (*g*) Migrating white storks *Ciconia ciconia*; flocking enables knowledge transfer between generations and remote visual detection of thermals through observation of others. Arrows indicate features of interest described in legend. Original images by Graham Taylor, except (*b*) by Kate Reynolds.

The use of flying robots is growing rapidly across a diverse range of applications. Current applications include goods delivery, environmental monitoring, infrastructure inspection, search and rescue, and reconnaissance and surveillance [1]. Unfortunately, poor endurance and an inability to operate in windy or turbulent conditions present bottlenecks for many flying robots [2]. Yet, birds manage to soar flexibly in a wide range of challenging conditions (figure 1*a*), demonstrating the existence of many untapped opportunities for harvesting atmospheric energy in natural and anthropogenic landscapes. These everyday opportunities for atmospheric energy harvesting are of clear technological relevance, given that the range and endurance of a small uncrewed air system (sUAS) is limited by its onboard energy capacity [3]. Most sUAS employ electric propulsion, despite the low specific energy (up to 0.9 kJ g^−1^) of state-of-the-art lithium battery technologies [4], compared with the high specific energy of the fat-based energy stores (37.6 kJ g^−1^ wet mass) that birds metabolize over most of the duration of their long endurance flights [5]. Atmospheric energy harvesting therefore has the potential to extend electric vehicle range and endurance substantially [2]. Even so, there have been few practical demonstrations of autonomous soaring to date [6–14], and all have been implemented on high-performance sailplanes flying in favourable conditions, rather than on operational sUAS flying in everyday environments. By contrast, birds including gulls, falcons, kites and crows can be seen soaring these environments opportunistically in a wide range of weather conditions, using a mixture of flapping and gliding flight and widely different flight morphologies (figure 1*a*).

Our goal in this review is therefore to identify avian soaring strategies whose translation to engineering could result in substantial improvements in the range and endurance of future sUAS. With this in mind, we do not set out to review specific algorithmic aspects of technical applications of autonomous soaring, which have been reviewed elsewhere in relation to gradient soaring [15] and thermal soaring [16,17]. Rather, we consider the extent to which technical research has yet explored the myriad opportunities for soaring that birds already exploit across the wide range of natural and anthropogenic (e.g. built) environments in which current and future sUAS may operate. Our overarching aim is therefore to inspire new avenues of research in autonomous soaring, by synthesizing the growing body of research on soaring birds. As we show, some of the most significant new opportunities may lie not in the strong updrafts and wind gradients that have been the focus of most technical and biological research to date, but in the everyday opportunities afforded by small-scale updrafts, turbulence and shear. To demonstrate this, we review what is known of the soaring strategies of birds, with the aim of identifying key themes, knowledge gaps and missed opportunities. We begin by elaborating the mechanical energy flows associated with soaring flight (§2), which provides a formal basis for analysing the full scope of these opportunities. We then consider the wide-ranging atmospheric conditions that birds exploit for soaring, their associated design considerations and their relationship to the state-of-the-art in autonomous soaring (§§3–5). Finally, we identify new prospects for autonomous flight in relation to sensing systems, control algorithms and path planning, exploring which specific opportunities are being missed, and how these can be exploited (§6).

## 2. Energetics of atmospheric energy harvesting

It can be shown from first principles that the mass specific flow of aerodynamically useful mechanical energy (d*e*/d*t*) in soaring flight is that given by equation (2.1) below (see [18,19] and electronic supplementary material, Text for derivation). Our review is therefore structured around the several distinct opportunities for atmospheric energy harvesting that this equation identifies. Equation (2.1) shows that the opportunity to harvest atmospheric energy in flight exists if either: (i) the wind has a mean upward component; or (ii) the wind varies spatially or temporally. Both circumstances are likely to co-occur, but whereas the wind supplies gravitational potential energy when energy is harvested from an updraft (which is called static soaring), it supplies aerodynamic kinetic energy when energy is harvested from a spatio-temporal wind gradient (which is called dynamic soaring). Each of these distinct sources of atmospheric energy is well known, but writing them out explicitly provides the following complete and succinct expression:

2.1 $$\frac{de}{dt}=\frac{\overset{\begin{array}{c} \mathrm{drag}\;\mathrm{losses}\\ \mathrm{net}\;\mathrm{of}\;\mathrm{thrust} \end{array}}{\overbrace{(T-D)V}}}{m}+\overset{\mathrm{static}\;\mathrm{soaring}}{\overbrace{gW_{U}(s,t)}}-\overset{\mathrm{dynamic}\;\mathrm{soaring}}{\overbrace{\left[V\cdot \frac{\partial W}{\partial t}+V\cdot \frac{\partial W}{\partial s}\frac{ds}{dt}\right]}},$$

which may be used to elaborate all the opportunities that birds and sUAS can exploit for harvesting atmospheric energy. Here, the air velocity ***V*** of the bird/vehicle and the wind velocity ***W*** are both defined in an Earth-fixed axis system, where *W_U_* is the upward component of ***W***. The first term on the right-hand side represents the aerodynamic losses due to drag (*D*) net of thrust (*T*) at a given airspeed V=‖V‖, expressed relative to body mass (*m*). These aerodynamic losses are offset by the energy harvested from the atmosphere in soaring flight, and we do not consider them further here. The second term represents the mass specific energy flow due to static soaring, which is the product of the gravitational acceleration (*g*) and local updraft speed *W_U_*(*s*, *t*), expressed in terms of a path coordinate (*s*) and time (*t*). Section 3 focuses upon the exploitation of static soaring by birds, distinguishing their energetically equivalent but behaviourally different use of thermal versus orographic updrafts. The final two terms within the square brackets split the total specific energy flow due to dynamic soaring, −V⋅dW/dt, into components arising from the explicit and implicit time-dependence of the wind when traversing a spatio-temporally varying wind field. These two parts describe gust soaring and gradient soaring respectively, which are distinct but energetically equivalent forms of dynamic soaring.

## 3. Static soaring: exploiting updrafts

The specific energy flow associated with static soaring, *gW_u_*(*s*, *t*), is positive if the wind has an upward vertical component (*W_u_* > 0) and negative if it has a downward component (*W_u_* < 0), so energy is gained in an updraft but lost in a downdraft. Somewhat surprisingly, perhaps, this specific energy flow is independent of any morphological properties of the bird or vehicle itself. Hence, while we tend to associate static soaring with high-aspect ratio wings and low wing loading (see §3.3 below), these features of a bird's flight morphology serve to enable the efficiency, rather than occurrence, of atmospheric energy harvesting. The ability to exploit static soaring ultimately hinges on the ability to find and remain within the strongest region of an updraft, which may be achieved either by planning a path which biases the expected vertical component of the wind upward, or by reacting appropriately to the local wind conditions. Either way, the principle is the same: dwell in updrafts and avoid downdrafts.

### 3.1. Opportunities for static soaring

**Figure 2.**
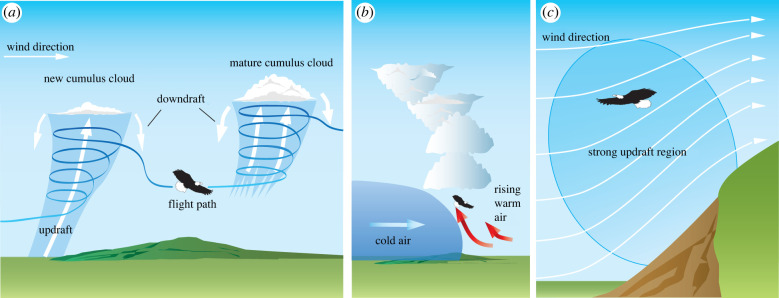
Opportunities for static soaring in updrafts. (*a*) Soaring in thermals using typical circling flight behaviour. (*b*) Soaring ahead of cold fronts. (*c*) Soaring in orographic updraft on windward side of landscape feature.

Atmospheric updrafts can occur in one of two ways, leading to the two distinct types of static soaring: thermal and orographic. Thermal soaring is possible in the buoyant updrafts that occur because of warming of the air close to the Earth's surface (figure 2*a*, left), convection at the base of clouds (figure 2*a*, right), or the intrusion of a cold front into a mass of warmer air (figure 2*b*) [20]. Orographic soaring is possible where an updraft is created when winds are obstructed by ridges, buildings, cliffs, ships, waves or vegetation (figure 2*c*). Both kinds of soaring are quite predictable and have been used to explain the routes that birds follow, on spatial scales ranging from local [21–24] to continental [25–28]. Thermal-soaring behaviour is usually favoured over orographic soaring by land-soaring birds such as eagles [29], which may delay their migration until weather conditions suit, but will switch to using orographic updrafts when time is of the essence [30]. This reflects the fact that thermal soaring offers greater flexibility over the routes that a bird can take over land [31], and perhaps also more updrafts available to exploit in the heat of the day. More generally, land-soaring birds display a flexible exploitation strategy that mixes thermal and orographic soaring according to the local conditions [21,23,25,26,30,32,33]. A similarly flexible approach to the use of thermal versus orographic updrafts is likely to be important in land-soaring sUAS, and both sources of updraft may also be accessible at sea.

Most studies of avian static soaring consider specialized land-soarers such as vultures whose form is adapted to flying in updrafts strong enough to sustain gliding flight [34,35]. In practice, most soaring birds also use flapping, and the world's heaviest soaring bird, the Andean condor *Vultur gryphus*, is considered noteworthy in being a static-soaring species that almost never flaps its wings [36]. This presumably reflects the fact that the same energetic principles apply in powered and unpowered flight (equation (2.1)), such that many birds exploit weak updrafts using a combination of both gliding and flapping flight. For example, the common kestrel *Falco tinnunculus* specializes in wind-hovering over natural and anthropogenic landscape features (figure 1*b*; electronic supplementary material, Movie S1) [37,38], hanging on fixed wings in strong updrafts, but interspersing flapping with gliding in weak updrafts [39]. Other species that glide when possible will often flap in weak thermal updrafts [40–46], or in orographic updrafts produced by small-scale features [21] and weak winds [47]. Larger soaring birds sometimes use flapping for control rather than thrust production, tucking their wings in response to turbulence, in a motion resembling one half of a flapping cycle [48,49]. Nevertheless, these observations raise the intriguing possibility that many other birds that we do not conventionally think of as soaring species, such as domestic pigeons *Columba livia domestica*, may in fact be benefitting routinely from the energetic gains provided by flapping in updrafts. Models of the energetics of flapping flight have not in general considered the energetic subsidy that updrafts provide [46], which may be an overlooked feature of the energetics of commuting and migration in species using flapping flight.

In summary, birds of various forms and sizes opportunistically exploit updrafts, using a mixture of powered and unpowered flight. It follows that robotic vehicles could make similarly flexible use of soaring opportunities when monitoring anthropogenic structures that generate their own updrafts [50–52], or when flying specific routes within an urban environment where updrafts are prevalent [33]. Indeed, a coastal development with a reliable prevailing wind might even be designed to promote such opportunities [22]. Such behaviours could be integral to the flight of almost any atmospherically aware robot, as even a hovering multi-rotor positioning itself in the updraft on the windward side of a building will reduce the vertical thrust that it requires [53]. The key opportunity that we see is for small flying robots of all kinds, whose operation at low altitudes within the atmospheric boundary layer creates opportunities for exploiting updrafts, coupled with an inherent risk of encountering downdrafts. Similar opportunities are present on other planets with an atmosphere, so need not be limited to Earth-bound robots, given the recent success of NASA's Ingenuity helicopter on Mars [54].

### 3.2. Atmospheric aspects of static soaring

#### 3.2.1. Thermal soaring

A thermal updraft comprises a rising mass of air that is warmer than the surrounding air, and hence thermally buoyant (figure 2*a,b*). Thermals vary considerably in size and intensity—up to thousands of metres in height and width, with updraft speeds from 1–2 m s^−1^ [55] to 5 m s^−1^ [56]. Columnar thermals have a strong convective core rising in a continuous stream up from the ground [57] and are typically associated with a weaker surrounding downdraft (figure 2*a*, left) and sometimes also with a weaker updraft. By contrast, bubble thermals comprise a toroidal vortex ring structure of limited vertical extent that itself rises upward like a bubble in a fluid [57], such that while the updraft through the centre of the vortex ring may be strong, the air beneath it might not be rising at all (figure 2*a*, right). Because thermals result from atmospheric temperature differentials caused by uneven solar heating of the Earth's surface, thermal formation depends on local ground cover and local weather conditions. These arise in particular locations on timescales lasting from seconds to hours [26]. Where thermals are triggered at regular intervals by solar heating of specific ground features, wind drift can cause a regularly spaced thermal street to form [20,29].

Soaring birds usually circle close to the thermal core, thereby maximizing potential energy gain and inter-thermal glide range in cross-country flight [20,29,58]. Soaring birds have also been found to optimize turn radius by varying bank angle [59] or airspeed [60] as a function of altitude to compensate for altitudinal variation in thermal size and air density. However, in regions where thermals are abundant, and particularly where thermal streets occur, birds may pass straight through a thermal without circling [20,29,33], demonstrating flexibility in the behavioural strategies that they use to soar. This is called dolphin-style flight [61] and maximizes the speed at which ground is covered, rather than maximizing potential energy gain. Birds using dolphin-style flight slow down when they sense a thermal [29] to increase the potential energy gained as they transit it, and fly faster in static or sinking air to minimize their losses [20]. However, it is likely that the presence of visible cues in the form of a cloud street could facilitate proactive path planning in dolphin-style flight. Circling and dolphin-style flight are both well known to glider pilots, so thermal-soaring sUAS could presumably use a similarly flexible range of behaviours to exploit thermal updrafts [62]. Of course, the requirement to circle within a thermal core may be avoided by hovering rotorcraft, which could potentially exploit thermals to attain altitude faster and with lower energy consumption than would otherwise be required in a powered climb—either by hovering within the thermal, or using dolphin-style flight through it.

Thermals are strongest on land, where they form only during the day, but weaker thermals may form at sea at any time of the day or night owing to the thermal stability of oceanic surface waters. This phenomenon is exploited by frigatebirds *Fregata* spp. (Figure 1*c*) [55], which may soar to altitudes of greater than 4000 m on the open ocean by using the weak thermal updrafts found beneath cumulus clouds [63] and the stronger updrafts within them [44,64]. Some raptors also make opportunistic use of thermal lift over the sea on migration [45,65], and flocks of gulls *Larus* spp. have been observed using coordinated low-altitude circling to trigger thermal formation in the unstable boundary layer at the sea's surface [66]. Levant sparrowhawks *Accipiter brevipes* have been reported to use the weak thermal updrafts caused by heating of tarmac along roads in desert environments for linear low-altitude thermal soaring on migration [67], and common swifts *Apus apus* have been reported soaring the thermal updrafts associated with sea breeze fronts [68]; figure 2*b*. Nocturnal thermal soaring is not usually possible on land, but has been reported in turkey vultures *Cathartes aura* (figure 1*d*) over flared methane vents at a landfill site [69], and may be possible in some other anthropogenic environments with flaring such as oil fields. In summary, although the principles and practice of strong-thermal soaring are well understood by glider pilots, birds offer insights into a range of novel weak-thermal soaring behaviours [70] that sUAS could potentially exploit. The utility of these (and other) novel atmospheric energy sources will of course depend upon the extent to which they provide useful energy gain in relation to the costs or constraints associated with exploiting them.

#### 3.2.2. Orographic soaring

An orographic updraft is produced when a horizontally moving air mass is deflected upward over topographical features or other structures in the environment (figure 2*c*). The strength, size and shape of the updraft are therefore influenced by the strength and direction of the wind in relation to the size, shape and orientation of the obstacle. In contrast with thermal updrafts, which can be thousands of metres in height over the oceans, orographic updrafts tend to be relevant only at lower altitudes relative to the ground. Slopes, ridges, dunes, cliffs, mountains and other elevated terrain generate strong and predictable orographic updraft, so much of the research on orographic soaring concerns how large soaring birds such as eagles exploit this on migration [26,71]. Much less is known of how this reliable energy source is used in everyday flight behaviours, but soaring raptors routinely use orographic updraft in the mornings when thermals have not yet developed [72], and gulls will routinely soar the windward sides of buildings [22], drainage dykes [21] and ships [73], and switch to using orographic updraft in conditions unfavourable for thermalling [33]. So adept are they at exploiting local updrafts that gulls are even able to soar small-scale landscape features such as roads and tree lines [24]. The accompanying turbulence makes this a challenge, but may be actively exploited by some specialist soarers such as turkey vultures that also fly along tree lines [74]; see below. Gulls flying ahead of seafront buildings have been found to favour parts of the wind field in which the effects of gusts are reduced [22]. By making use of such small-scale structures generating orographic updraft, birds may significantly reduce the energetic costs of flight during their daily activities, so it would clearly make sense to exploit the same opportunities in sUAS during low-altitude operation. For example, seabirds have learned to exploit the updrafts created by ship structures, and the potential of this opportunity for maritime patrol vehicles has recently been demonstrated for a sUAS soaring the updraft of a ship at less than 5% throttle [75].

Waves are the only natural source of orographic updraft on the open ocean and can generate updrafts even in the absence of wind, owing to the wave's own progression relative to the air. Their relatively small scale limits the maximum achievable increment in potential energy, so instead of gaining significant altitude, pelagic birds such as albatrosses, fulmars, gulls and pelicans typically soar in ground effect just above the surface (figure 1*f*) on the windward side of long, steady waves [56,76]. This wave-slope soaring behaviour, called sweeping flight [77], is observed along moving waves even in zero wind [76,78] and allows the bird to increase glide speed without losing altitude, by using the supply of potential energy obtained through static soaring to offset the higher drag losses incurred at higher airspeeds [56]. This elevated glide speed can be converted to potential energy when reaching the end of the wave, thereby allowing the bird to glide against the wind to restart the process on another wave [77]. This unconventional form of static soaring could be accessible to maritime sUAS including small ekranoplans (i.e. vehicles designed to fly in ground effect, typically over the sea). Common swifts *Apus apus* have also been recorded performing a behaviour resembling sweeping flight in the thermal updrafts present along sea breeze fronts [68], which they seek out in the same way as human glider pilots.

### 3.3. Design considerations in static soaring

#### 3.3.1. Flight morphology

Because the specific energy flow in static soaring, *gW_u_*(*s*, *t*), depends only on updraft strength (equation (2.1)), any bird or vehicle can realize energetic gains from flying in an updraft. Nevertheless, the ability to exploit such flows to glide long distances depends on having a sink rate no greater than the updraft speed. In gliding, sink rate scales as the square root of wing-loading, so lower wing-loadings facilitate exploitation of weaker updrafts [34]. Other things being equal, sink rate will also be lower for a higher aspect ratio wing, because of its higher lift-to-drag ratio, so higher aspect ratio wings can also facilitate exploitation of weaker updrafts [34,35]. Both features are taken to an extreme in frigatebirds, whose ability to exploit weak thermals over the open oceans [44] is explained by their exceptionally low wing-loading and high aspect ratio [55,63] relative to other thermal-soaring birds (figure 1*c*). In principle, powered flight can be used to remain in an updraft weaker than the airframe's sink rate, where the potential benefits will depend on the extent to which the energy gained exceeds the cost of obtaining it. Even so, because aerodynamic power requirements scale as mass times the square root of wing-loading [34], birds or vehicles with lower wing-loading will always be better able to make use of weaker updrafts in powered flight.

Gaining energy effectively in an updraft is only one element of cross-country soaring, which also involves gliding quickly and efficiently between updrafts. This means having a high best glide speed, defined as the groundspeed attained at minimum glide angle in still air, which depends on having a high aspect ratio wing and a reasonably high wing-loading [34]. This is also important in sweeping flight along ocean waves (figure 1*f*), so species that make use of this technique have long slender wings enabling high-speed flight with a shallow glide angle [77]. For species such as gulls, which combine thermal and orographic soaring with flapping, wing form is likely to be a compromise in terms of wing-loading and aspect ratio. Specifically, the intermediate aspect ratio wings of gulls give good glide performance, but with lower power requirements during flapping than a higher aspect ratio wing would provide, which makes them well suited to switching efficiently between flapping and soaring flight modes [33]. Another potentially conflicting design objective is the need to turn tightly to exploit smaller, weaker thermals. Minimum turn radius scales linearly with wing-loading [34,55], so for a given best glide speed, the broad wing chord of a typical land-soaring bird confers a tighter minimum turn radius than the narrower wings typical of sea-soaring birds [34]. There is therefore a trade-off between straight and turning flight performance, which will need to be optimized at the design stage in soaring sUAS.

Different species of bird appear to be optimized for different updraft intensities suited to their own specific flight characteristics [29]. Turkey vultures (figure 1*d*), for example, have a 30% lower wing-loading than black vultures (figure 1*c*) of similar body mass, which allows them to ascend in weaker thermal conditions and hence earlier in the day [79]. Conversely, it makes them less well suited to covering long distances after leaving a thermal, which may at least partly explain why they remain closer to the ground than species with higher wing-loadings [74,79]. Specific food-searching strategies may influence this preference, as flying closer to the ground may help in sensing odour cues versus relying on visual cues at higher altitudes. Generally, larger birds are more likely to make use of thermal updrafts than smaller ones [34]—presumably because they acquire disproportionately more energetic benefit from soaring owing to the adverse scaling of aerodynamic power requirements with body mass [29]. These effects can be quite subtle: even within a species, the flight behaviour of the larger sex can be more strongly influenced by environmental conditions affecting updraft availability [80].

It is an open question whether similar scaling principles will hold true of sUAS. Most technical research has considered vehicles with wingspans greater than 1.5 m, which limits atmospheric energy harvesting to regions where strong thermals are prevalent, or to localities with reliable orographic updrafts. Smaller vehicles with lower wing-loading should be able to exploit smaller, weaker updrafts, but their lower flight speed would also be expected to increase their sensitivity to turbulence [81]. Gust tolerance may therefore set a lower practical size limit for robotic soaring, depending on the turbulence of the environment in which the vehicle must operate. Conversely, an upper size limit is likely to be set by the higher sink rate of a larger aircraft in combination with its lower manoeuvrability. Even so, the success of crewed sailplanes demonstrates that in appropriate conditions, updrafts can be used by larger aircraft to soar on flights of hundreds to thousands of kilometres.

#### 3.3.2. Sensory systems

**Figure 3.**
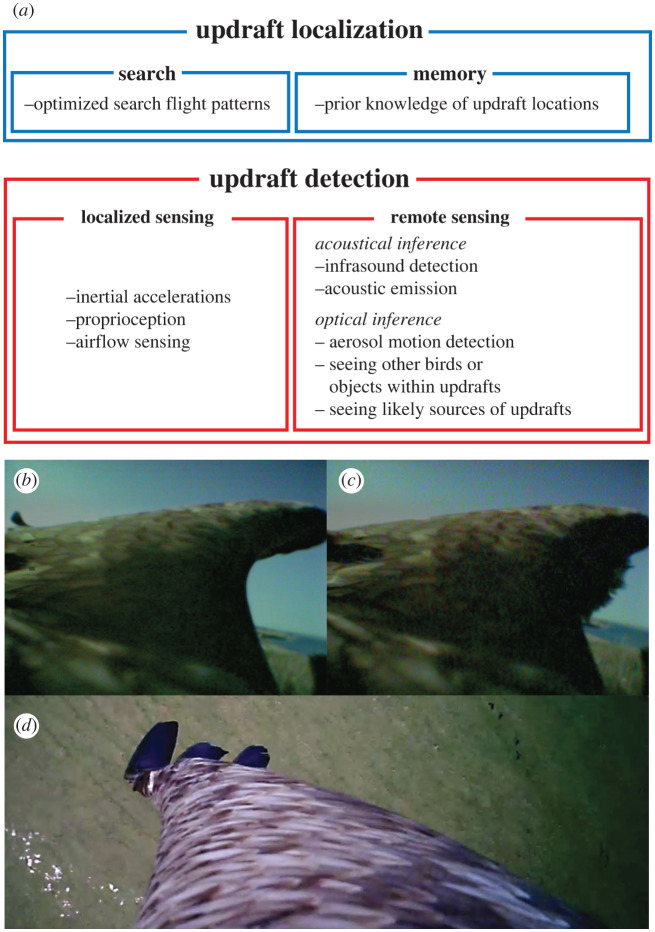
Possible mechanisms of updraft detection and localization by birds. (*a*) Hypothesized mechanisms of updraft detection. (*b–d*) Onboard video along wing of a steppe eagle *Aquila nipalensis* soaring orographic updrafts on sea cliffs [85]. (*b,c*) Aeroelastic deflection of covert feathers at wing leading-edge as bird encounters clifftop updraft; de-interlaced video frames at 0.06 s separation. (*d*) Aeroelastic deflection of wingtip primary feathers when turning.

To exploit updrafts, birds must first locate them. Birds almost certainly remember where updrafts occur [82], and social species such as storks [83] and vultures [58] may pass this knowledge to their offspring through demonstration—particularly on their first migration (figure 1*g*). It is also likely that birds can predict updraft formation through experience, as vultures have been found to adapt their decisions on when to depart thermals on the basis of recent experience of the prevailing conditions [84]. Among the mechanisms of updraft detection that have been proposed (figure 3*a*), vision is the only one that birds use remotely. Like human glider pilots, birds detect thermal updrafts at a distance by observing other birds circling within them [29,66,86–88]. Social species such as vultures [87] and storks [88] use this for collective sensing [29,89,90] (figure 1*g*), flying faster between thermals if they have sight of another individual in a destination updraft [87]. Remarkably, even species which do not usually soar (e.g. common starling *Sturnus vulgaris*) have been observed to climb successfully in a thermal when a soaring species is present as a guide [86]. Soaring birds also use growing cumulus clouds to identify the location of thermals [29,63] and will sometimes enter clouds to climb [29,64]. It is likely that they use the presence of haze to detect the presence of sea breeze fronts [68], and it has even been speculated that birds can see updrafts by observing heat shimmer or aerosol movement [82], although there is no direct evidence to support this. Birds can sense atmospheric infrasound at frequencies from 0.5 to 10 Hz [91] and may be able to locate its source by detecting Doppler shifts [92], which could be used for remote detection of updrafts, weather fronts and wind interacting with terrain, but again this has not been tested empirically. The sensory mechanisms by which birds may predict or remotely detect updrafts clearly warrant further research. For instance, we may speculate that birds learn to predict updrafts by observing the association between energy gain and terrain (e.g. flying towards ridgelines expecting orographic updraft, or bare hillsides expecting thermals).

Birds can almost certainly detect updrafts directly as they enter them. The otolith organs of the inner ear are able to detect linear accelerations [93,94], which are felt as an upward surge on entry to an updraft and may also be used for thermal centring. Birds can detect small changes in air pressure over short periods, probably by using the paratympanic organ in the middle ear [95], so may be able to track changes in altitude through the accompanying barometric pressure variation. Visual cues such as optic flow may provide an alternative mechanism for enabling sensing of the altitude gain [96] in a thermal, although this is likely to be less sensitive than acceleration/force-based cues except when flying close to the surrounding terrain. Again, the sensory mechanisms by which birds detect updrafts as they encounter them warrant further research.

Little is known of the behavioural mechanisms that birds use to remain circling within a thermal core, but specialist thermal-soaring species have presumably evolved reactive control mechanisms to do so, probably by sensing differences in loading across the wings or the roll motions that result from these. For example, the roll moment resulting from the difference in the aerodynamic forces on the two wings has been shown to provide sufficient information to enable good thermal-soaring performance when combined with information on vertical acceleration [97]. Proprioception, defined as the ability to use muscle strain receptors to sense the pose of body parts and the forces acting on them, therefore represents another mechanism by which birds may sense updrafts. Birds can also sense changes in the airflow over their wings using mechanoreceptors near the feather follicles [98]. Local flow reversal deflects the covert feathers (figure 3*b,c*) so mechanoreceptors at their base may sense flow separation or changes in angle of attack [85,99]. The alula feathers forming a leading-edge flap (figure 1*b*), and primary feathers forming the slotted wingtips (figure 3*d*), each sustain strong aerodynamic loads in updrafts that can presumably be sensed mechanically. Feather mechanoreceptors also respond to vibration, which may be important in sensing airspeed and flow separation. Visual copying behaviour may also be used to remain within a thermal if a suitable visual guide is present.

## 4. Dynamic soaring: exploiting gradients and gusts

Equation (2.1) shows that the total specific energy flow due to dynamic soaring, −***V*** · d***W***/d*t*, is positive whenever the wind velocity ***W*** changes in opposition to the velocity ***V*** of the bird or vehicle relative to the air. This outcome can be achieved by flying windward in a wind whose speed is increasing through time, or leeward in a wind whose speed is decreasing through time, which facilitates cyclical dynamic soaring wherever there is a reliable gradient or step-change in wind speed [100]. Aerodynamic kinetic energy will be lost if the windward–leeward phasing of flight is reversed. Hence, whereas static soaring is conditional on being in the right place at the right time, dynamic soaring is conditional on moving at the right velocity for a given point in space and time. The phasing of the kinetic energy flows in dynamic soaring will appear different if the kinetic energy is defined with respect to the inertial speed of the bird/vehicle, rather than its airspeed. Nevertheless, despite earlier controversy in the literature, these two perspectives are now agreed to be equivalent [101]. Considering the flow of aerodynamic kinetic energy, as in equation (2.1), focuses attention on the spatial and temporal wind gradients that are essential to dynamic soaring.

### 4.1. Opportunities for dynamic soaring

The opportunity for dynamic soaring exists whenever the wind speed varies in space (which is the basis of gradient soaring) or time (which is the basis of gust soaring). While gradient soaring and gust soaring exploit distinct opportunities, they are energetically equivalent (equation (2.1)). Hence, as the wind field is rarely constant and never homogeneous in the atmospheric boundary layer, we should typically expect to find mixed exploitation of both kinds of dynamic soaring. Moreover, because thermal and orographic updrafts are predictably associated with wind shear and turbulence, we should further expect the static-soaring strategies of birds to be adapted to make additional gains through gust and gradient soaring.

Because aerodynamic kinetic energy can be obtained through dynamic soaring in powered as well as unpowered flight, pelagic birds that engage in dynamic soaring when the wind is strong will often flap their wings when the wind is weak [19,102–104]. Nevertheless, the great majority of research on avian dynamic soaring has focused on albatrosses [77,78,105–110], which are capable of flying immense distances without flapping, and whose flight morphology is highly specialized for this function [111]. Moreover, because it is challenging to demonstrate empirically which sources of atmospheric energy are being employed in situations where a bird uses flap-gliding flight, or where gradients and updrafts occur together (as they do whenever waves are present), the use of gradient soaring has only been confirmed in albatrosses and (more recently) shearwaters [19]. Nevertheless, for the reasons discussed below, dynamic soaring can be assumed to be prevalent across a much wider range of species than this. Harvesting energy from spatio-temporal wind gradients therefore has great potential for increasing the range and endurance of a variety of sUAS, because surface shear is present whenever there is a wind, and its occurrence is not necessarily tied to specific landscape features. This makes dynamic soaring practical close to the Earth's surface, without having to deviate too far from a path planned to meet other mission requirements. For this reason, autonomous dynamic soaring is now a highly active field of research [112], although the great majority of studies to date have been done in simulation rather than in technical implementation.

### 4.2. Atmospheric aspects of dynamic soaring

#### 4.2.1. Gradient soaring

**Figure 4.**
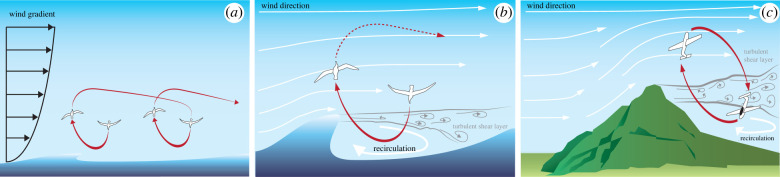
Opportunities for dynamic soaring in spatially varying wind fields. (*a*) Gradient soaring within the thick shear layer over the sea's surface. (*b,c*) Gradient soaring across the thin shear layer that peels off from a breaking wave (*b*) or sharp ridge (*c*) and which separates fast-moving air above from slow-moving recirculating air below.

The aerodynamic interaction of wind and terrain creates spatial wind gradients with predictable structure. Such gradients occur within the thick shear layer forming the atmospheric boundary layer (figure 4*a*), and across the thin shear layer that peels off the crest of a sharp ridge or breaking wave, separating the fast-moving air above from the still air in its lee (figure 4*b,c*). Both phenomena are used by pelagic birds to glide great distances without flapping, and the largest albatrosses have been tracked flying using this technique for over 13 days at 950 km per day [111]. Spatial gradients are also ubiquitous in well-developed turbulence and may therefore be used by birds for gradient soaring [74], but as these gradients are usually unpredictable, they effectively blend with gust soaring. Equation (2.1) shows that the specific energy flow associated with gradient soaring is

$$-V\cdot \frac{\partial W}{\partial s}\frac{ds}{dt},$$

where *s* is a path coordinate. Hence, because the wind is expected to be slower closer to a surface, the general principle of flying windward in a wind of increasing speed and leeward in a wind of decreasing speed can be restated more specifically for gradient soaring as climbing windward and descending leeward [100]. An alternating windward rise and leeward fall is therefore diagnostic of gradient soaring [18,108,110,113,114], usually involving an undulating flight path whose sinuosity is expected to vary according to how the overall travel direction relates to the overall wind direction [106,107,109]. This phasing of the horizontal and vertical components of flight can be used to identify dynamic soaring even in cases where the details of the wind field may be unknown [19].

Trajectory optimization in gradient soaring is a complex problem that typically involves detailed modelling of how the flight dynamics interact with the wind field. Most studies assume a logarithmic wind profile, which implies that most of the kinetic energy gain will occur in the region of maximum shear close to the surface [115]. However, this is also where waves and turbulence interfere most strongly with the assumed wind profile, which may explain why these models do not closely predict the flight trajectories recorded in albatrosses, which follow shallower arcs than expected [115]. Even so, it is possible to draw some quite general conclusions about trajectory optimization for dynamic soaring without the need for a detailed model of the wind gradient. For a horizontal wind field with local vertical shear gradient *σ* ≥ 0, such as can be expected over a flat surface, we may rewrite the specific energy flow associated with gradient soaring as follows:

4.1 $$-V\cdot \frac{\partial W}{\partial s}\frac{ds}{dt}=-V^{2}\sigma \cos\gamma \sin\gamma \cos\eta ,$$

where *γ* is the aerodynamic flight path angle defined as the elevation angle of the bird's air velocity ***V*** with respect to the horizontal, and where *η* is the heading-to-wind angle defined as the angle between the horizontal wind vector and the horizontal component of the bird's air velocity [19]. It is clear by inspection that the rate of energy harvesting is proportional to the quantity $\epsilon_{h}=-\mathrm{sgn}\gamma \cos\eta$ , called the horizontal wind effectiveness [19], where the signum function sgnγ=±1 is positive when ascending and negative when descending. The rate of energy harvesting is therefore maximized by aligning ascent and descent through the shear layer as closely as possible with the windward and leeward directions, respectively. It follows that the opportunity for gradient soaring is greatest when the overall direction of travel is across the wind, because then the kinetic energy that is gained on the upwind and downwind legs can be used to enable crosswind progression. Gradient-soaring Manx shearwater *Puffinus puffinus* have been found to bias their outbound foraging journeys in a crosswind direction [19], and Bulwer's petrels *Bulweria bulwerii* have also been found to have a strong preference for crosswind progression when foraging in North Atlantic trade winds [116]. The utility of gradient soaring to marine sUAS will therefore depend upon the extent to which progress must be made in other directions relative to the wind.

Other observational studies have indicated that pelagic birds exploit the rapid increase in wind speed encountered when climbing out of the leeward eddy behind a tall wave [56,105,107], rather than the less-rapid increase in wind speed encountered above a flat surface. In this case, most of the harvested energy is obtained impulsively when flying across the thin shear layer that results from the wind flow separation over the crest of an obstruction, rather than continuously when flying through the thick atmospheric boundary layer. This behaviour is close to the step model envisaged in Rayleigh's original description of dynamic soaring [100] and has been exploited by remote-controlled sailplane pilots to achieve speeds in excess of 244 m s^−1^ in the lee of sharp ridges during repeated looping manoeuvres [113,117]. Detailed observations of the gradient-soaring behaviours of pelagic birds could therefore play an important role in developing gradient-soaring strategies for sUAS [15,118]. This could include both the initial specification and subsequent training of a controller adapted using machine learning. Machine learning of gradient soaring has only been undertaken in simulated environments [119–123], so the real-world performance of the resulting controllers is only as good as the assumptions they learn on the spatio-temporal structure of the wind field. Directly measuring the wind environment via flying robots is in its infancy with only limited work using anemometers affixed to sUAS (e.g. [124,125]). Studying bird behaviour, on the other hand, provides a proven starting point for learning to fly in challenging real-world environments [126], particularly if inertial measurements of the bird's flight behaviour are combined with local airspeed measurements made using a Pitot tube [18].

#### 4.2.2. Gust soaring

**Figure 5.**
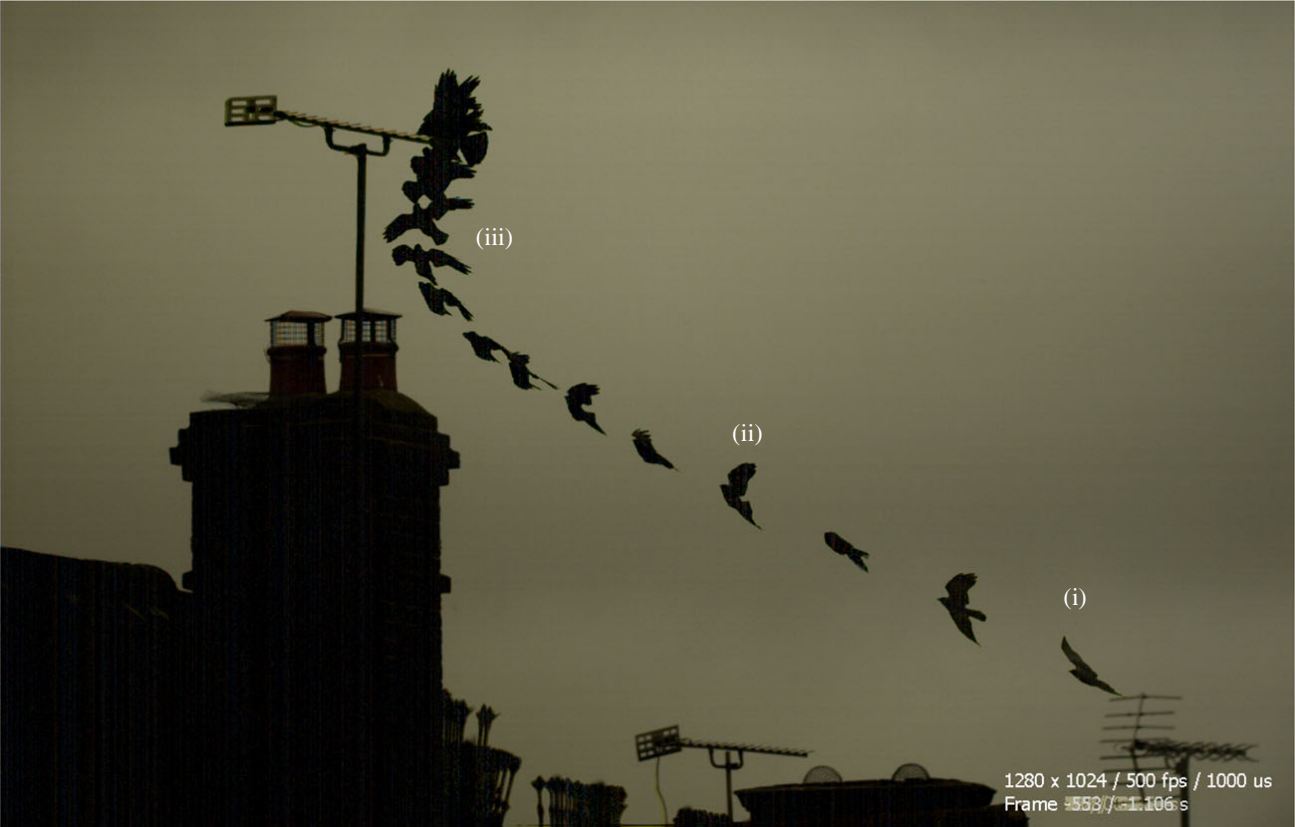
Mixed mode soaring in a spatio-temporally varying wind field. Composite image from the video of a Eurasian jackdaw *Corvus monedula* landing into the wind; 0.1 s timesteps shown. The bird is shown: (i) making a left-handed turn into the wind; (ii) entering a shallow climb in the lee of the building and (iii) climbing almost vertically as it exits the lee of the building and soars the strong headwind and presumed updraft it encounters. Turbulence apparent from the bird's movements (ii) is consistent with transit through a separated shear layer. Original video by Graham Taylor; see electronic supplementary material, Movie S4.

Gust soaring has been much less well studied in birds, which reflects the difficulty of measuring or predicting the turbulence that they encounter locally. Nevertheless, birds can often be seen performing flight manoeuvres that cannot be explained by any other mechanism [18,74,113,127]. This is most obvious when a bird that is making slow progress against a strong headwind suddenly gains altitude (figure 5), often wheeling downwind at very high groundspeed. This gain of altitude must reflect the conversion of a sudden increment in aerodynamic kinetic energy into an increase in gravitational potential energy through a transient increase in lift production, as described by the specific energy flow

$$-V\cdot \frac{\partial W}{\partial t}\;$$

that is associated with gust soaring (equation (2.1)). Moreover, turning downwind after encountering a sudden headwind provides the opportunity to harvest further atmospheric energy as wind speed decreases in any subsequent lull.

Opportunistic gust-soaring behaviours are most commonly observed in birds that specialize in gliding flight close to the ground (figure 5), including crows [18,113], swallows and martins [127] and various raptors [74]. Larger species such as turkey vultures follow a characteristically contorted flight path when gliding at low altitudes. This is thought to be linked to the exploitation of shear-induced turbulence generated at the edge of forested areas—particularly when weather conditions are unfavourable for the formation of thermals [74]. A similar rocking flight pattern is displayed by the bateleur eagle *Terathopius ecaudatus* [128], which has also been suggested to make use of temporal variation in the wind field to subsidize its flight costs [29]. Rocking flight behaviours are typical of some other raptors that fly close to the ground, including harriers *Circus* spp., so this may be a more widespread strategy again. In any case, the regularity of such flight behaviours makes them obvious candidates for application in gust-soaring sUAS. Even so, the growing body of research on autonomous dynamic soaring currently focuses on predictable wind gradients [112] rather than unpredictable gusts (but see [3,129]).

### 4.3. Design considerations in dynamic soaring

#### 4.3.1. Flight morphology

Equation (2.1) shows that whereas the specific energy flow due to static soaring, *gW_u_*(*s*, *t*), depends only on the local updraft strength *W_u_*(*s*, *t*), the specific energy flow due to dynamic soaring, −***V*** · d***W***/d*t*, varies in proportion to airspeed V=‖V‖. Hence, whereas a bird that is transiting an updraft may benefit from reducing its airspeed in order to maximize its total gain in potential energy [29], a bird that is transiting a gust or shear layer will obtain no such benefit. On the contrary, for a given rate of change in wind speed d*W*/d*t*, the rate at which energy is gained through dynamic soaring increases with airspeed *V*. Furthermore, because dynamic soaring can only subsidize a significant proportion of a bird's flight costs under windy conditions, a high airspeed may be necessary simply to enable control over the bird's flight track. It follows that while the same principles of efficient aerodynamic design apply in both static and dynamic soaring, always favouring wings of high aspect ratio, higher wing-loadings may be expected in birds that specialize in dynamic soaring, favouring narrower wings at a given mass. This is broadly the pattern that is observed when comparing obligate land-soaring and sea-soaring birds [34], and in some species, the flight behaviour of the sex with the higher wing-loading is more strongly influenced by wind conditions favourable for dynamic soaring [130].

The mechanisms by which birds tolerate gusts depend on sensing flow disturbances and responding appropriately [16], or mitigating gusts by having a flexible, morphing airframe of appropriate geometry [17–20]. Birds that use gust or gradient soaring to loiter close to the ground tend to have very low wing-loading. This may relate to their need to be highly manoeuvrable to respond quickly to unpredictable turbulence in a structured environment. Some of these species also display strong wing dihedral, which is typical of turkey vultures [74] and harriers, whose characteristic rocking flight may be promoted by the high static roll stability that their strong wing dihedral provides [131,132] (figure 1*d*). High static stability may also be important in aligning a bird's flight into a gust. Domestic pigeons gliding in urban environments also make extensive use of wing dihedral—presumably for similar reasons. Other species such as swallows *Hirundo* spp. make active use of a forked tail to control turning flight manoeuvres associated with rapid altitude gain [127]. Indeed, several species that apparently specialize in gust soaring, including the red kite *Milvus milvus* and swallow-tailed kite *Elanoides forficatus* (figure 1*e*), also possess prominently forked tails which they twist and spread for control.

#### 4.3.2. Sensory systems

Although birds that rely on dynamic soaring may use prior knowledge to optimize where and how they fly [133], harvesting atmospheric energy from spatio-temporal gradients requires an ability to sense wind direction, which in turn requires airflow sensing to be combined with an inertial or visual sense of progression relative to the ground. Many of the sensing mechanisms that have already been mentioned for updraft detection, including inertial sensing, flow sensing and proprioception (figure 3), appear suitable for detecting wind gradients. In addition, petrels and albatrosses are reported to possess a specialized organ for detecting changes in airspeed, in the form of tubular nostrils hypothesized to function as Pitot-static tubes [105,134]. This may be a key adaptation for gradient soaring, as these are the only families of birds that routinely use this mode of flight, and the only families of birds to possess such a structure. Any such sense of airflow also needs to be referenced to an inertial frame of reference to determine wind direction. This may be achieved visually, as gulls flying low over water have been found to regulate translational optic flow (i.e. the apparent relative motion of the surface as they progress over it) in a manner that would allow them to control their altitude coupled to their groundspeed [96]. A view of the horizon provides information on bank angle [135], which may be useful in controlling dynamic soaring trajectories at sea. It may also be possible for birds to predict the location of spatial gradients based on the landscape features that generate them, such as the crests of hills and waves (figure 4*b,c*). It therefore seems reasonable to assume that birds combine visual, aerodynamic and inertial information to achieve dynamic soaring.

## 5. State of the art in autonomous atmospherically aware soaring

**Figure 6.**
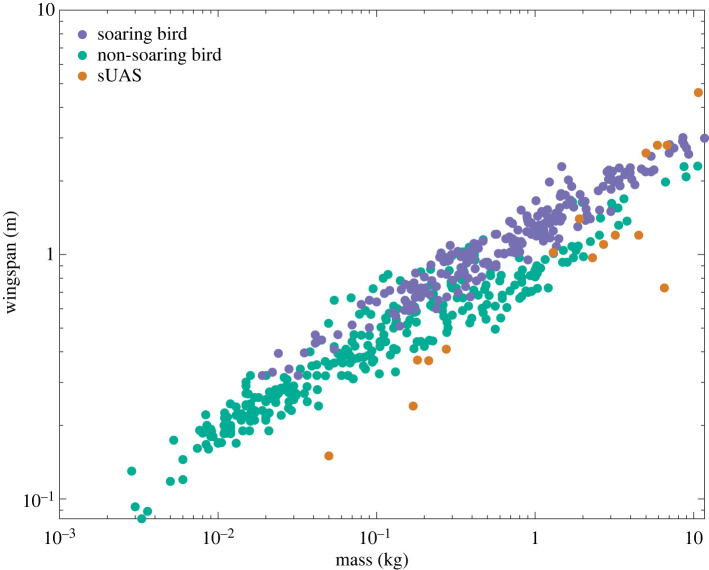
Scaling of soaring and non-soaring birds and operational sUAS. Within the sample of *n* = 507 birds shown, the *n* = 209 species recorded as soaring (purple) tend to have a larger wingspan for a given body mass than the *n* = 298 species that have not been recorded as soaring (green). The *n* = 16 different models of operational sUAS (orange) tend to have a smaller wingspan than birds of similar body mass, except at the very highest masses. See text for discussion. Bird dataset collated from published sources [34]; sUAS dataset collated by Abdulghani Mohamed; see electronic supplementary material, Data.

As we have discussed above, extracting useful mechanical energy from the atmosphere does not in itself necessitate any specific flight morphology, so even a hovering rotorcraft could benefit from the lower power requirement associated with flying in a steady updraft. Nevertheless, it is reasonable to assume that fixed- or morphing-wing vehicles operating at a similar range of aspect ratio and wing-loading to birds will be best placed to capitalize upon the full range of soaring opportunities that birds exploit. These metrics are not always openly available for operational sUAS, but it is clear from figure 6 that for a given mass, operational sUAS tend to have a shorter wingspan than birds—especially those species that have been recorded as soaring. It is difficult to draw strong conclusions regarding this difference in the spans of birds and sUAS, because their speed regimes are often different. It is likely, for example, that the feathered, articulated, musculoskeletal wing structures of birds are better able to cope with gusts and aeroelastic flutter than the fixed-wing structures of conventional sUAS, permitting a greater span at a given mass (and hence at a given wing-loading, or a given airspeed). As an exception that illustrates the rule, the Airbus AlbatrossONE demonstrator uses hinged wings that deflect freely in gusts to enable a longer, higher aspect ratio wing than would be possible with a conventional fixed-wing design [136].

Other bioinspired wing designs may prove important to realizing the full potential of autonomous soaring, given both the need for gust tolerance and the likely benefits for soaring of having longer wings than are deployed in current sUAS. For example, the use of wing dihedral for enhanced lateral stability versus a forked tail for enhanced lateral control (figure 1*d,e*) appear to represent alternative—and opposing—strategies to responding appropriately to turbulence (see §4.3.1 above). This may be an interesting dichotomy to explore in the context of sUAS designed to operate within the turbulent conditions found close to the ground. For instance, simulation studies have shown that building lateral instability into the airframe of an autonomous vehicle can promote gust tolerance, with a lower control demand than a vehicle with lateral stability would experience under gusty conditions [137]. More generally, the use of avian-inspired morphing-wing designs could enhance dynamic soaring manoeuvres [138], through the enhanced gust tolerance [136,139] and flexible flight performance that morphing wings provide (electronic supplementary material, Movies S1–S4). Both features are important to birds for optimizing flight in the non-uniform and variable wind fields that are essential to dynamic soaring [122,138]. In other respects, autonomous soaring is fundamentally a problem in guidance and control, which can in principle be solved using novel sensors and algorithms on existing airframes. Implementing autonomous soaring in a current or future flying robot therefore hinges upon making it atmospherically aware.

Most opportunities for autonomous soaring can be exploited through reactive flight control (e.g. turning to remain in an updraft) or proactive path planning (e.g. flying to the windward side of a structure) [27]. The specific rules that birds follow to soar are largely unknown, but autonomous static soaring has been successfully demonstrated from first principles in sUAS resembling high-performance sailplanes using reactive flight control. For example, inertial sensing of an uncommanded roll motion indicates higher lift on the ascending wing, typically resulting from the presence of an updraft that can be exploited by rolling back towards that side. It is plausible that new capabilities could be acquired in a black-box fashion by using supervised machine-learning approaches in which birds serve as expert demonstrators. However, as the algorithmic aspects of technical soaring have already been reviewed at length elsewhere [15–17], we have chosen to focus our attention here upon identifying other promising avenues for exploration at the interface of biology and engineering.

### 5.1. Thermal soaring

Autonomous thermal soaring has been successfully demonstrated using sUAS resembling high-performance sailplanes [6–12,14,16], for which the main challenges are locating a thermal, and then flying a trajectory that harvests its energy in an efficient manner [17]. Local thermal detection and autonomous thermal centring has proven successful in sUAS flying between preprogrammed waypoints: having detected a thermal directly, it is possible to fly an efficient trajectory using an explicit thermal-centring algorithm that makes use of inertial sensing of roll motion and acceleration as described above [6–12,140–142]. Other attempts have involved algorithms that plan a flight path by weighing near-field updraft velocity estimates [143], or dynamically mapping thermal updrafts [16,17]. Black-box algorithms acquired through reinforcement learning have also been reported [62,97]. These successful demonstrations have all been achieved using conventional position, attitude, acceleration and airspeed sensors. The key outstanding challenge for autonomous thermal soaring is therefore locating distant thermals. This requires integration of information across a variety of sources—from long-range vision to models of elevation and terrain—in addition to prior experience. As in flocking birds (figure 1*g*), this problem may be most effectively solved by multiple atmospherically aware agents serving as a distributed sensor network [144]. The group soaring behaviours of flocking birds may therefore offer insight into efficient behavioural algorithms for information sharing when exploiting spatially complex energy resources such as thermals [87–90,144]. For example, in addition to merely enabling detection of a thermal, observation of the movements of other individuals can provide information on its size, strength and drift speed. It may be possible to infer the use of such information by observing the decisions that birds take when choosing when and where to enter or depart an occupied thermal, particularly in cases where there may be more than one thermal to choose between. Likewise, sUAS could benefit from using visual observations of soaring birds to identify, locate and classify thermal updrafts.

### 5.2. Orographic soaring

Radio-controlled glider pilots routinely fly in orographic updrafts, but autonomous orographic soaring is an emerging research area, with only a few flight trials to prove its feasibility [50,145]. Several recent studies modelling the flows around medium-rise buildings have concluded that atmospheric energy extraction is possible under stable atmospheric conditions [50–52,145–149]. Other features that generate updrafts adequate for soaring with model aircraft, and hence with sUAS, include hedges, tree lines and walls. Orographic soaring could therefore be useful in low-altitude operations in complex urban environments, which are rich with updraft-generating obstacles, and might also be useful for traversing long distances at very low altitudes to remain below radar. On the other hand, in adversarial scenarios such as urban warfare, there may be a trade-off between the energetic benefits of soaring in orographic updrafts, and the resulting predictability of the agent's behaviour.

For reactive flight control of a single sUAS, conventional inertial and airspeed sensors can be used to detect updrafts [70], but bird-inspired airflow sensing using sensors distributed across the wing [150–152] or mounted to sense upstream flow effects [124,125] could also enhance updraft detection and tracking. However, travelling between static updrafts is challenging in urban environments owing to their structural complexity, the presence of moving obstacles and the occurrence of large gusts [145]. There is therefore likely to be a trade-off between the energetic benefits of soaring in orographic updrafts in cluttered environments, and the risk of a collision. Past experience can also assist prediction of updraft locations using real-time mapping [153] or prior modelling [50,145,149] of the wind field. Observations of where birds position themselves when flying through urban environments [22] could therefore be used by sUAS to provide real-time feedback on updraft locations and could also be used offline as expert demonstrations for supervised learning of path-planning algorithms. Moreover, flying robots could use swarm-level communication to enhance their collective search for updrafts in urban settings without the constraint of having to maintain line-of-sight as birds must do when observing each other.

### 5.3. Gradient soaring

Implementing autonomous dynamic soaring is made challenging by the difficulty of detecting favourable wind conditions in an inherently complex flow field, and by the precise flight manoeuvres required for energy harvesting. Simulation studies have shown that energy-neutral trajectories are possible for sUAS flying in the thick shear layer found close to the Earth's surface and across the thin shear layers behind ridge tops [154], but the precise manoeuvres that these require are made challenging by turbulent conditions [155]. Gradient soaring may involve either closed or open-circuit flight trajectories [15,138,156,157], but both require relatively large spaces to manoeuvre. Closed-circuit flights have been achieved using radio-controlled recreational models [158] and more recently by autonomous vehicles [159]. These exploit the shear layer formed over a spine-backed ridge where the flow up the windward side of the hill meets the rotor flowing up the leeward side of the hill (figure 4*c*). Energy is gained in a substantial lump when encountering the prevailing wind at the transition across the shear layer. However, wing flutter is a significant issue during the associated transition from a strong tailwind to a strong headwind. The aerodynamic loads can be several times those due to gravity, which requires an airframe capable of bearing these high stresses within its weight limit. In the light of these constraints, morphing-wing designs analogous to those of birds have been suggested as a solution for optimized energy extraction [15,138]. Open circuits are ideal for long-distance travel, but the literature describing these is currently confined to simulation studies [15,114,160,161]. Real-world implementation of open circuits is currently constrained by inadequate sensor measurements, limited computation, and the challenge of estimating a dynamic wind field, but would enable atmospherically aware sUAS to progress along a line of buildings, hedge line, or waves in a maritime setting, while extracting energy to stay aloft.

### 5.4. Gust soaring

Autonomous gust soaring is even more challenging given the randomness and short timescales of atmospheric turbulence [162]. Controllers which enable energy to be harvested from gusts have been demonstrated [129,163], and conventional inertial and airspeed sensing and a barometer were found to be sufficient for gust soaring given the assumptions used in these modelling studies. There may also be advantages to resolving the direction of the wind. Flush air data systems, such as those inspired by albatross nostrils [164], or multi-hole pressure probes [125,129] can provide both wind speed and direction which could enhance gust-soaring performance. Some work has been done to implement gust mitigation controllers on robotic aircraft [81,124], which will be important in minimizing attitude perturbations during gust soaring.

### 5.5. Opportunities for atmospherically aware path planning

**Figure 7.**
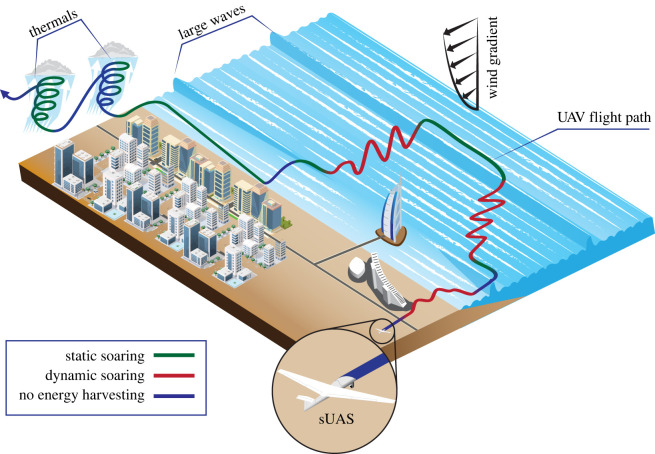
Opportunities for bird-inspired mixed mode soaring by sUAS. Urban developments, particularly in coastal regions with predictable winds or thermals, could be planned to create these opportunities.

Although energy-efficient atmospherically aware path planning has been considered in the context of extending range or endurance [7,8,149,165–182], it has not, to our knowledge, been implemented in realistic mission scenarios or missions. The study that has come closest to modelling a realistic scenario is described in a pair of papers modelling a cooperative mission by multiple sUAS required to overfly a specific point of interest at an airfield [174,175]. Numerous controllers have been proposed and validated for autonomous thermal soaring [6–9,142,172,182–189], and a smaller number of studies have proposed controllers for autonomous shear soaring [13,119,190,191] and autonomous gust soaring [163,183,184,191–194]. These existing controllers and control architectures could already form the basis of a soaring-capable flight controller embedded in a realistic mission simulation model. So too could proposed methods of estimating wind field state for thermal soaring [195] and gust soaring [196,197]. Unsurprisingly, simulation studies of small powered sUAS confirm that thermal soaring could result in a pronounced improvement in flight endurance under favourable atmospheric conditions in open environments [12,198], but similar benefits have also been demonstrated by a simulation study of planned and opportunistic autonomous thermal soaring in a model designed to simulate atmospheric conditions in Northern Europe [172]. Furthermore, the flexible mixed-strategy approach observed in birds such as gulls, kites and crows, combining different kinds of dynamic and static soaring has not yet been formally described, let alone implemented in any flying robot (figure 7). Likewise, algorithms capable of classifying a wide variety of atmospheric phenomena and thereby enabling sUAS to select the appropriate harvesting strategy from among a mixed set are yet to be explored.

### 5.6. Regenerative soaring

One key opportunity available to sUAS, but not to birds, is the use of windmilling regenerative systems to charge onboard electrical cells [199,200]. No analogue of the dynamo appears in nature, just as there is no analogue of the wheel, so our engineered systems should be able to surpass biological ones in this respect. Closed-circuit soaring flight trajectories have also been explored for ground-based electricity generation, where controlled flight manoeuvres are conducted to gain kinetic energy in order to spin a generator on the ground through a tether [201]. This tethered flight concept has been demonstrated successfully on small vehicles, with plans to build larger ones capable of accessing larger wind gradients at higher altitudes so as to produce megawatts of electricity [202]. Another separate strand of research has successfully combined autonomous thermal soaring with solar photovoltaic technology to achieve regenerative flight durations in excess of 26 h [203].

## 6. Conclusion

Birds can be seen soaring in many natural and anthropogenic environments where future sUAS could usefully operate. Nevertheless, whereas the thermal, orographic and gradient-soaring behaviours of specialist soaring species have been widely studied, surprisingly little is known of their mechanisms of proactive path planning and reactive flight control, or of the sensors that they engage for these purposes. Moreover, with only a few exceptions, the current literature does not address the opportunistic gust-soaring behaviours and other forms of high-frequency low-gain energy harvesting that can be observed in a wide range of species using flap-gliding flight in turbulent air. Even so, birds offer many examples confirming that soaring is possible in a far wider range of environments and conditions than are currently accessible to, let alone exploited, by current sUAS. If there is a useful source of atmospheric energy to exploit, birds are likely to have exploited it, so simply observing where and when they choose to fly offers a good source of inspiration for the kinds of strategy that future sUAS might mimic. This throws up some genuine surprises, including the maritime thermals exploited nocturnally by frigate birds, or the flared methane vents exploited nocturnally by turkey vultures at landfill sites. It also reveals some more obvious—but still unexploited—opportunities, like surfing gusts on windy days, soaring the updraft on a ship or other maritime structure, or sweeping close to the sea's surface on the updraft of a long wave. Beyond the scope of this review, the study of other flying animals such as bats and insects is relevant to identifying further opportunities for atmospheric energy harvesting, particularly under nocturnal conditions [204], and similar opportunities for soaring can exist within aquatic environments, as demonstrated by swimming sharks' use of tidal updrafts [205].

Exploiting the many transient opportunities that birds exploit, whether through static or dynamic soaring, may require the development of sUAS possessing comparable gust tolerance to birds, which could in turn necessitate the use of flexible, morphing-wing designs. In other respects, the energetics equations show that no uniquely specialized flight morphology is needed to extract useful energy from the atmosphere (see equation (2.1)). What is needed is rather to be flying in the right place, at the right time, with the right velocity and orientation. This in turn explains why birds which do not share the specialized soaring flight morphology of albatrosses and vultures can still soar opportunistically where the potential to do so exists. This provides assurance that powered aircraft, which may not look like high-performance sailplanes, can still benefit from soaring energy gains. Just as a bird can obtain gravitational potential energy in a thermal while flapping, even a multi-rotor can gain harvest atmospheric energy through static soaring. Hence, while there is obvious scope for highly specialized sUAS that soar the oceans like albatrosses, or which make persistent use of thermals like vultures, the greatest potential may lie in drones that make flexible use of local updrafts and spatio-temporal wind gradients (figure 7). New research on the soaring strategies of birds, particularly concerning the mechanisms of sensing and individual and collective behaviour that they use to implement these, has much to offer current and future flying robots.

**Box 1. Glossary of terms.**

term: **meaning**
static soaring: harvesting of atmospheric energy by flying in an updraft
dynamic soaring: harvesting of atmospheric energy by flying in a spatially or temporally varying wind field
thermal soaring: static soaring involving flight in a thermal updraft
orographic soaring: static soaring involving flight in an orographic updraft
sweeping flight: orographic soaring using the updraft created by the movement of a wave at sea
gradient soaring: dynamic soaring involving flight in a spatially varying wind
gust soaring: dynamic soaring involving flight in a temporally varying wind
regenerative soaring: soaring of a powered vehicle with harvested atmospheric energy used to recharge a battery
updraft: a rising mass of air
thermal: an updraft driven by the buoyancy of a mass of air warmer than the surrounding air
thermal street: a line of thermals created by downwind convection of thermals from their point of origin
atmospheric boundary layer: an air mass whose motion is slowed by friction with the Earth's surface
wind shear: a spatial wind gradient including that occurring in the atmospheric boundary layer
ground effect: the reduction in aerodynamic drag associated with flight just above the Earth's surface
sUAS: small uncrewed air system (or systems)
wing-loading: the ratio of mass to wing area
aspect ratio: the ratio of wing span to wing mean chord (i.e. wing span squared divided by wing area)
sink rate: a flyer's rate of descent with respect to the surrounding air mass

## Data Availability

The morphological data needed to recreate figure 6 are provided as electronic supplementary material, Data S1. The original video data used to create figure 1*b* and figure 5 are provided as electronic supplementary material, videos S1 and S2 [206].
